# Urgent lung volume reduction surgery is effective for secondary pneumothorax in emphysema patients

**DOI:** 10.1093/icvts/ivad099

**Published:** 2023-06-08

**Authors:** Annalisa Barbarossa, Dirk Van Raemdonck, Walter Weder, Laurens J Ceulemans

**Affiliations:** Department of Thoracic Surgery, UZ Leuven, Leuven, Belgium; Department of Chronic Diseases and Metabolism, BREATHE, KU Leuven, Leuven, Belgium; Department of Thoracic Surgery, UZ Leuven, Leuven, Belgium; Department of Chronic Diseases and Metabolism, BREATHE, KU Leuven, Leuven, Belgium; Department of Thoracic Surgery, Bethanien Klinik, Zürich, Switzerland; Department of Thoracic Surgery, UZ Leuven, Leuven, Belgium; Department of Chronic Diseases and Metabolism, BREATHE, KU Leuven, Leuven, Belgium

**Keywords:** Chronic obstructive pulmonary disease, Emphysema, Lung volume reduction surgery, Pneumothorax

## Abstract

Secondary pneumothorax due to emphysema can be life-threatening and requires surgery in most situations. Here, we extended lung resection to close the fistula using lung volume reduction surgery (LVRS). We present a patient with chronic obstructive pulmonary disease and secondary spontaneous pneumothorax referred after ineffective treatment by chemical pleurodesis. Urgent LVRS followed by elective LVRS was performed obtaining air-leak resolution and significantly improving pulmonary function and quality of life. We discuss the surgical technique and effectiveness of LVRS as a treatment for pneumothorax.

## INTRODUCTION

Chronic obstructive pulmonary disease (COPD) is a major cause of chronic morbidity and mortality worldwide [1]. Lung volume reduction surgery (LVRS) has gained an important role as a treatment option in well-selected patients [2]. However, in case of life-threatening complications like secondary spontaneous pneumothorax (SSP), the extent of lung resection using LVRS can be challenging and is rarely discussed. We present the case and surgical technique of a successful urgent LVRS, which significantly improved the patient’s spirometry and functionality. An institutional reporting agreement was obtained: MP021523/KU Leuven.

## CASE REPORT

**Figure 1:**
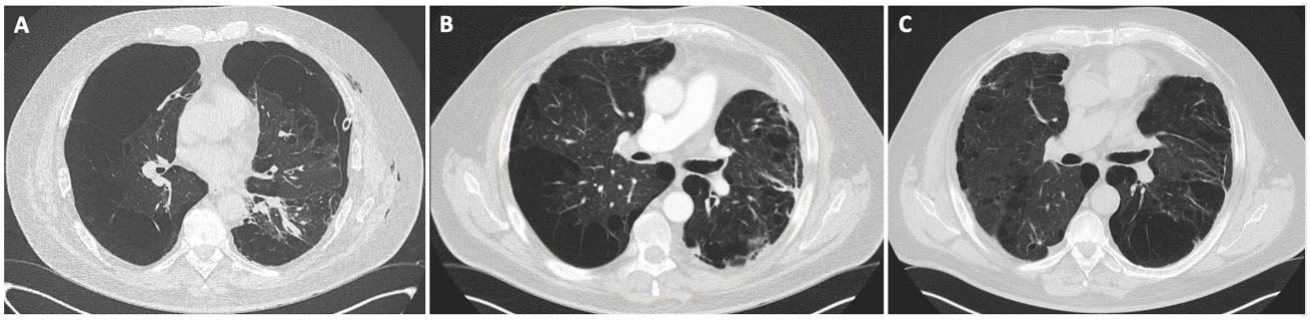
Chest CT following talc pleurodesis (**A**). Chest CT 1 month after left lung volume reduction surgery (**B**). Chest CT 1 month after right lung volume reduction surgery (**C**).

A 68-year-old male (35 packyear ex-smoker; BMI 38) with severe emphysematous COPD and obstructive sleep apnoea, no exercise tolerance and oxygen dependent, presented to the emergency room with acute shortness of breath. His last spirometry results, 5 months earlier, are summarized in Table 1. Chest X-ray showed a left-sided tension pneumothorax for which a chest drain was inserted. CT scan confirmed left-sided pneumothorax with limited reinflation of the lung and bilateral destroyed emphysema. After 11 days, the patient was discharged with Heimlich valve. Since the air leakage persisted, talc pleurodesis by VATS was performed 2 weeks later (Fig. 1A). Three weeks later the chest tube was removed and the chest X-ray showed residual pneumothorax. The same day, the patient was readmitted and chest CT showed increased pneumothorax with severe adhesions. A drain was reinserted and the patient was referred to an academic institution.

**Table 1:** Functional evaluation from first admission to last follow-up, spirometry with body box plethysmography

	5 months before admission to ER	6 months after left LVRS	6 months after right LVRS	3 years after bilateral LVRS	4 years after bilateral LVRS
FEV1 (% predicted)	44	48	60	60	59
RV (% predicted)	211	138	N/A	88	86
TLC (% predicted)	124	103	101	98	97
DLCO (% predicted)	35	44	51	62	59
6MWD (m)	422	480	467	389	413
BMI	38	34	38	40	39.7

LVRS: lung volume reduction surgery.

An urgent three-port VATS was performed. After identification of the air leak and extensive adhesiolysis, a unilateral LVRS was performed (Reinforced-Signia™, Medtronic, Minneapolis, USA), having the lungs semi-inflated. The lung was covered with Neoveil™ (polyglycolic acid, Gunze, Tokyo, Japan). The patient was admitted to the intensive care unit with 2 chest tubes. Twelve days later, he was discharged with Heimlich valve, followed by total resolution of air leak and lung expansion within 2 weeks (Fig. 1B). Following ambulatory rehabilitation and medical therapy (triple inhalation; corticosteroid, long-acting antimuscarinic agent and long-acting beta2-agonist) optimalization, the patient showed significant functional improvement (Table 1). Eight months later, the patient was completely recovered and an elective contralateral LVRS was performed (three-port VATS). He was discharged 4 days later and reinitiated the ambulatory rehabilitation, resulting in further functional improvement (Table 1). A decrease in hyperinflation was confirmed on chest CT (Fig. 1C). At the last follow-up, 3 years after initial admission, a continued spirometry and functional improvement was observed, despite weight regain (explaining the decreased walking distance) (Table 1).

## DISCUSSION

SSP is a frequent event, which can be attributed to 78% to COPD [3]. The most common treatment is chest drainage or talc pleurodesis without lung resection, resulting mostly in prolonged air leakage and hospitalization [4]. However, if the patient presents with heterogeneous or even homogeneous emphysema, surgical evaluation should be considered [5]. In contrast to an elective LVRS, patients with SSP cannot undergo the same work-up, compromising selection criteria (active smoker, no rehabilitation, no recent chest CT or spirometry), resulting in a more stringent LVRS approach by limiting the volume that can be resected and performing a unilateral intervention. First, the air leak should be located by intermittent ventilation and immersion of the lung under water. Once found, an extensive adhesiolysis should be performed—if needed—the lung should be properly recruited and based on CT scan analysis (if available), the most destroyed lung tissue can safely be resected. Most fragile lung parts can be covered by tissue reinforcement.

An urgent surgical treatment of the pneumothorax using the LVRS concept will not only increase the chance of resolving the air leak in the short term, but it has the potential to improve the pulmonary function in the long term. Furthermore, after the patient has recovered from the procedure, and the COPD is properly optimized from a medical point of view, an elective LVRS on the contralateral side can be considered.

Therefore, patients with COPD and SSP should be referred, after placement of a chest drain, to a centre with experience in multidisciplinary COPD evaluation and LVRS.

## Data Availability

The data underlying this article are available in the article.

## References

[1] Halbert RJ , Natoli JL, Gano A, Badamgarav E, Buist AS, Mannino DM. Global burden of COPD: systematic review and meta-analysis. Eur Respir J 2006;28:523–32. 16611654 10.1183/09031936.06.00124605

[2] Martinez FJ , Flaherty KR, Iannettoni MD. Patient selection for lung volume reduction surgery. Chest Surg Clin N Am 2003;13:669–85. 14682601 10.1016/s1052-3359(03)00101-7

[3] Hallifax RJ , Goldacre R, Landray MJ, Rahman NM, Goldacre MJ. Trends in the incidence and recurrence of inpatient-treated spontaneous pneumothorax, 1968-2016. J Am Med Assoc 2018;320:1471–80. 10.1001/jama.2018.14299 PMC6233798 30304427

[4] Varela G , Jiménez MF, Novoa N, Aranda JL. Estimating hospital costs attributable to prolonged air leak in pulmonary lobectomy. Eur J Cardiothorac Surg 2005;27:329–33. 15691691 10.1016/j.ejcts.2004.11.005

[5] Nava GW , Walker SP. Management of the secondary spontaneous pneumothorax: current guidance, controversies, and recent advances. J Clin Med 2022;11:1173. 35268264 10.3390/jcm11051173 PMC8911306

